# Detection and Molecular Characterization of GI-1 and GI-23 Avian Infectious Bronchitis Virus in Broilers Indicate the Emergence of New Genotypes in Bolivia

**DOI:** 10.3390/v16091463

**Published:** 2024-09-14

**Authors:** Doris Villanueva-Pérez, Luis Tataje-Lavanda, Angela Montalván-Avalos, Diego Paredes-Inofuente, Suly Montoya-Ortiz, Gisela Isasi-Rivas, María F. Fernández, Manolo Fernández-Sánchez, Manolo Fernández-Díaz

**Affiliations:** 1 Research and Development Laboratories, FARVET, Carretera Panamericana Sur N° 766 Km 198.5, Chincha Alta 11702, Peru; luis.tatajel@upsjb.edu.pe (L.T.-L.); angela.montalvan@farvet.com (A.M.-A.); d.paredes@farvet.com (D.P.-I.); s.montoya@farvet.com (S.M.-O.); gisasi@farvet.com (G.I.-R.); maferzfc@gmail.com (M.F.F.); manoloj@farvet.com (M.F.-S.); 2 Escuela Profesional de Medicina Humana, Universidad Privada San Juan Bautista, Lima 15067, Peru

**Keywords:** Infectious Bronchitis Virus, broiler, Bolivia, GI-1 lineage, GI-23 lineage, S1, phylogeny

## Abstract

Infectious Bronchitis Virus (IBV) is a major threat to the poultry industry worldwide, causing significant economic losses. While the virus’s genetic structure is well understood, the specific strains circulating in Bolivia have remained uncharacterized until now. This study aimed to identify and characterize new IBV strains in Bolivia. Tissue samples from broilers exhibiting clinical signs of Infectious Bronchitis were screened to detect IBV using real-time RT-PCR (RT-qPCR). Positive samples with low cycle threshold (Ct) values were selected for sequencing the full S1 gene. Of the 12 samples analyzed, 10 were determined to be positive for IBV. However, only four samples yielded sufficient genetic material for sequencing and subsequent phylogenetic analysis. The results revealed the presence of GI-1 and GI-23 lineages, both belonging to genotype I (GI). The GI-1 lineage showed >99% sequence identity to the H120 and Massachusetts vaccine strains, suggesting a close relationship. In contrast, the GI-23 lineage clustered with other IBV strains, showing a distinct subclade that is genetically distant from Brazilian strains. No evidence of recombination was found. Furthermore, amino acid substitution analysis identified specific mutations in the S1 subunit, particularly in the hypervariable regions 1, 2, and 3. These mutations could potentially alter the virus’s antigenicity, leading to reduced vaccine efficacy. The findings of this study highlight the importance of continued and broad genomic surveillance of circulating IBV strains and the need to improve vaccination strategies in Bolivia.

## 1. Introduction

Infectious Bronchitis (IB) is an acute, contagious disease affecting poultry worldwide, caused by the Infectious Bronchitis Virus (IBV) [1]. IBV is a highly contagious virus that affects multiple bird systems, including the respiratory, enteric, renal, and reproductive tracts [1,2,3]. Common symptoms include difficulty breathing, coughing, sneezing, and reduced egg quality and production [4,5]. IBV infection is the second most damaging poultry disease globally, following highly pathogenic influenza [6]. Live, live-attenuated, and inactivated vaccines, primarily derived from the Massachusetts (Mass) lineage 1 (GI-1), are used to protect and control poultry. However, vaccination provides incomplete protection due to IBV’s high capacity for genetic change through mutation, leading to the emergence of new IBV genotypes and variants [4,7]. Therefore, IBV requires continuous monitoring of its genetic composition to develop effective vaccines for specific regions.

IBV is a positive-sense single-stranded RNA (+ssRNA) virus classified within the family *Coronaviridae*, subfamily *Orthocoronavirinae*, and genus *Gammacoronavirus* [5]. Its approximately 27.6 kb sized genome encodes an RNA-dependent RNA polymerase and four structural proteins: nucleocapsid (N), membrane (M), envelope (E), and spike (S) [4,8,9,10]. The S protein consists of two subunits: S1, responsible for viral attachment to host cells, and S2, involved in viral entry. The S1 subunit contains hypervariable regions (HVR1, HVR2, and HVR3) that exhibit high diversity among different viral lineages and strains [9]. Moreover, the S1 subunit is responsible for inducing neutralizing antibodies and determining the virus’s antigenicity [11,12]. Currently, S1 genetic analysis is the gold standard for classifying IBV strains and assessing vaccine efficacy [10]. Therefore, the S1 gene phylogenetic analysis is crucial for identifying circulating IBV genotypes and lineages worldwide [4,13].

To date, nine IBV genotypes (GI to GIX) comprising 39 lineages have been identified worldwide [2,14,15,16,17,18,19]. Among these, lineages GI-1, GI-9, GI-11, GI-13, GI-16, and GI-23 have been reported in South American countries, including Colombia [20], Trinidad and Tobago [21], Uruguay [7,22], Chile [23,24], Peru [25,26], Argentina [7], and Brazil [2,9,27,28]. However, there are no available data on the circulation of different IBV lineages in Bolivia, hindering the development of effective control and vaccination strategies.

Most IBV outbreaks in South America involve the GI-1, GI-11, and GI-16 lineages. The GI-1 lineage is primarily associated with respiratory problems and is characterized as a vaccine-type strain [2,20,21,29]. The GI-11 and GI-16 lineages have been linked to respiratory disease, infertility, decreased egg production, and poor egg quality [30]. Some strains within these lineages have exhibited recombination events [7]. Recently, a new lineage, GI-23, was reported only in Brazil during an outbreak in 2022 [9] and deserves greater attention due to its rapid spread to different continents of the world [31,32]. The GI-23 lineage is of particular concern due to its high pathogenicity and association with severe upper respiratory and kidney diseases in poultry [2].

Despite vaccination against IBV in Bolivia’s high-density broiler areas, small outbreaks continue to occur primarily due to intensive farming practices. Recently, there has been an increase in cases of kidney and respiratory diseases associated with IBV infection in Bolivian broiler farms; however, the causative IBV strain remains unknown. To address this knowledge gap, this study aims to identify and characterize IBV strains affecting poultry production farms in Bolivia. A key contribution of this study is the first-time characterization of the full IBV S1 gene for the GI-1 and GI-23 lineages. This information could be used to improve vaccination programs and reduce recent outbreaks in Bolivia.

## 2. Materials and Methods

### 2.1. Clinical Samples

A total of twelve samples randomly collected from a commercial broiler were used in this study. The samples were collected from a poultry farm with suspected IBV infection located in Cochabamba, Bolivia, in April 2024. The samples were spotted on the 4 areas of the QIAcard FTA Classic (QIAGEN, Hilden, Germany) within 24 h of collection and dried for 3 h at room temperature. After drying, each sample-spotted FTA card was individually enclosed in double leak-proof zip-lock plastic bags. All samples were shipped to the Molecular Diagnostic Laboratory of Farmacologicos Veterinarios SAC (FARVET), Chincha Alta, Peru, and stored at room temperature until further processing. More detailed information on the samples is listed in Table 1.

### 2.2. RNA Extraction and Molecular Detection

All received samples were initially screened for IBV prior to virus sequencing. From each sample-spotted FTA card, 16 disks measuring 3 mm in diameter (4 disks per spotted area) were cut out using a sterile Harris Micro-punch 3.00 mm (Whatman, USA) and incubated for 30 min at room temperature in 400 µL of nuclease-free TE buffer (10 mM Tris-HCl; 0.1 mM EDTA, pH 8.0) to elute nucleic acids. Total RNA was extracted from 200 µL of the TE eluate using a QIAamp MinElute Virus Spin Kit (Qiagen, Hilden, Germany) on an automated QIAcube Connect instrument (Qiagen, Hilden, Germany) following the manufacturer’s instructions.

Detection was performed using RT-qPCR using primer sets IBV-N-F (5′-ATGCTCAACCTTGTCCCTAGCA-3′) and IBV-N-R (5′-TCAAACTGCGGATCATCACGT-3′) described by Meir et al. [33], targeting the Nucleocapsid (N) gene of the IBV genome. Amplification was performed using the Luna^®^ Universal One-Step RT-qPCR Kit (New England Biolabs, Ipswich, MA, USA) on a Rotor-Gene Q 5Plex HRM (Qiagen, Hilden, Germany), following the manufacturer’s instructions. The reaction mixture underwent reverse transcription at 55 °C for 10 min, initial denaturation at 95 °C for 1 min, followed by 40 cycles of denaturation at 95 °C for 10 s and annealing and extension at 60 °C for 30 s. The fluorescence signal of each amplified product was recorded continuously as the temperature was increased from 70 to 95 °C, with the acquisition of fluorescence data every 1.0 °C. This process amplified a 130 bp segment with a melting temperature (Tm) of 83–83.5 °C. Both the amplification curve (Ct) and melting curve (Tm) data were used to determine positive or negative status. Samples with low Ct values (Ct < 25), indicating high viral loads, were selected for full S1 gene amplification and sequencing analysis.

### 2.3. cDNA Synthesis and PCR Reaction for Full S1 Gene Amplification

Four positive IBV samples detected using RT-qPCR with Ct values < 25 were selected for full S1 gene amplification and sequencing (VFAR-187, VFAR-188, VFAR-189, and VFAR-190). For cDNA synthesis, 7 μL of total RNA (300 ng) from each sample and 2 μL of random hexamer primer (New England Biolabs, Ipswich, MA, USA) were subjected to reverse transcription using ProtoScript^®^ II Reverse Transcriptase (New England Biolabs, Ipswich, MA, USA) according to the manufacturer’s protocol. Subsequently, the full S1 gene was amplified using a reaction mixture containing 10 μL of Phusion Hot Start II High-Fidelity PCR Master Mix (1x) (Thermo Scientific, Waltham, MA, USA), 1 μL of forward primer (5′-TGAAACTGAACAAAAGAC-3′) (10 pmol/μL), 1 μL of reverse primer (5′-CCATAAGTAACATAAGGRCRA-3′) (10 pmol/μL) [34], 0.6 μL of DMSO, and 2 μL of cDNA in a final volume of 20 μL. PCR was performed on a 2720 Thermal Cycler (Applied Biosystems, Waltham, MA, USA) with the following conditions: 98 °C for 30 s, followed by 45 cycles of 98 °C for 15 s, 48 °C for 15 s, and 72 °C for 1 min, with a final extension at 72 °C for 5 min, resulting in a 1750 bp fragment. PCR products were visualized on a 1% agarose gel stained with SYBR Safe DNA gel stain (Invitrogen, Waltham, MA, USA) under Azure c600 Gel Imaging System (Azure Biosystems, Dublin, CA, USA). The amplified S1 gene PCR product was excised from the gel and purified using the QIAquick Gel Extraction Kit (Qiagen, Hilden, Germany) according to the manufacturer’s protocol. The DNA concentration was quantified using the Qubit^TM^ 1X dsDNA HS Assay Kit (Invitrogen, Waltham, MA, USA).

### 2.4. Library Preparation and Full S1 Gene Sequencing

The DNA library was prepared using 90 ng of each purified PCR product and the Rapid Barcoding Kit SQK-RBK114.24 from ONT (Oxford Nanopore Technologies, Oxford, UK). Native barcoding was employed for sample multiplexing, following the manufacturer’s protocol. PCR products were pooled in a 1:1 ratio using AMPure XP beads. After library preparation, 800 ng of the pooled sample was loaded onto a FLO-MIN114 R10.1 flow cell and sequenced on a MinION Mk1B device for 5 h under standard conditions. Real-time base calling was performed using Guppy (v6.5.7) embedded in the Mk1B with minKNOW software (v23.04.6).

The assembly process was conducted on the GalaxyTrakr platform [35] using default parameters. Quality control was assessed using FastQC (Galaxy Version 0.73 + galaxy0) [36], Porechop (Galaxy Version 0.2.4 + galaxy0) [37], NanoFilt (Galaxy Version 0.1.0) [38], and MultiQC (Galaxy Version 1.11 + galaxy1) [39]. Mapping was carried out with BWA-MEM (Galaxy Version 0.7.17.1) [40], followed by depth analysis using Samtools depth (Galaxy Version 1.15.1 + galaxy0) [41] and generation of the consensus sequence with Consensus Sequence (Galaxy Version 0.7.0 + galaxy1).

### 2.5. Recombination and Phylogenetic Analysis

Full S1 gene sequences were aligned with reference IBV strains from various lineages and genotypes isolated in South America and around the world, with sequences longer than 1000 nucleotides retrieved from GenBank. The alignments were performed using MAFFT version 7.526 [42,43], and phylogenetic trees were constructed using the Neighbor-Joining method with 1000 bootstrap replicates in MEGA version 11.0.13 [44]. The evolutionary distances were calculated using the Tajima–Nei method, and the rate variation among sites was modeled with a gamma distribution. This analysis involved 82 nucleotide sequences (Table S1) and 1416 positions after eliminating gaps and missing data. The analysis inferred the evolutionary relationships and displayed the optimal tree, with branch lengths reflecting the evolutionary distances. Additionally, recombination signals were assessed using the Recombination Detection Program version 5.34 [45] with default settings.

### 2.6. Amino Acid Substitution Analysis

Alignments of the amino acids of HVR1 (residues 38 to 67), HVR2 (residues 91 to 141), and HVR3 (residues 274 to 387) of the S1 gene belonging the GI-1 and GI-23 lineages were generated using MUSCLE in MEGA version 11.0.13 [44]. Key amino acid substitutions were visually inspected using CLC Genomics Workbench version 24.0 (QIAGEN, Germantown, MD, USA). The Israeli Variant 2 strain (AF093796.1) served as the reference for comparisons with the Bolivian strains VFAR-187 (PQ140481.1) and VFAR-189 (PQ140480.1), as well as with closely related strains from Poland 1251 (MZ666058.1) and Brazil A1 BRMSA 110/22-1 (OQ573556.1). The USA M41 strain (AY561711.1) served as the reference for comparisons with the other two Bolivian strains, VFAR-188 (PQ140483.1) and VFAR-190 (PQ140484.1), as well as the closely related strains from Trinidad and Tobago, 18RS1461-3 (MN696791.1) and Mexico IBV/ck/MEX/1616/19 (OM912698.1).

## 3. Results

### 3.1. RT-qPCR and Generation of Amplicons

**Figure 1:**
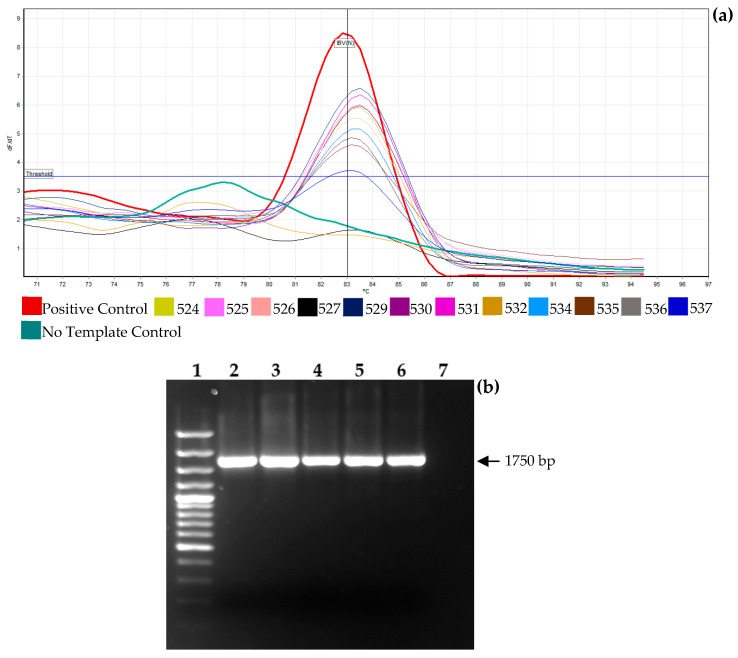
Detection of IBV from samples with suspected Infectious Bronchitis. (**a**) Melting curve analysis demonstrated specific amplification of the N gene in 10 of the 12 samples tested, indicating IBV positivity. Each sample is identified with a different thin line color; a Positive Control and a No Template Control were included. (**b**) Agarose gel electrophoresis of RT-PCR-amplified products confirmed the full S1 gene in the four positive samples with Ct values below 25. The arrow in the gel image indicates the amplified full S1 gene size (1750 bp). Lane 1: GeneRuler 100 bp Plus DNA Ladder, ready-to-use (Thermo Scientific, Waltham, MA, USA); lane 2: VFAR-187 (PQ140481.1); lane 3: VFAR-188 (PQ140483.1); lane 4: VFAR-189 (PQ140480.1); lane 5: VFAR-190 (PQ140484.1); lane 6: Positive Control; and lane 7: No Template Control.

Out of the 12 samples examined, 10 samples tested positive for the IBV N gene using RT-qPCR with a Tm value between 83 and 83.5 °C. The Ct values for these positive samples ranged from 18.93 to 31.92, indicating high levels of IBV RNA in some cases (Figure 1a). The VFAR-187, VFAR-188, VFAR-189, and VFAR-190 samples showed Ct values below 25 and were selected for full S1 gene sequencing and analysis. Specific primers were used to amplify the full S1 gene of IBV, and the expected (1750 bp) amplicon size was confirmed using 1% agarose gel electrophoresis (Figure 1b). Successful amplification of the full S1 gene was achieved for all four samples. Detailed information for each sample is presented in Table 1, including sample type, clinical signs, origin, year, and RT-qPCR Ct value.

### 3.2. Recombination and Phylogenetic Analysis

**Figure 2:**
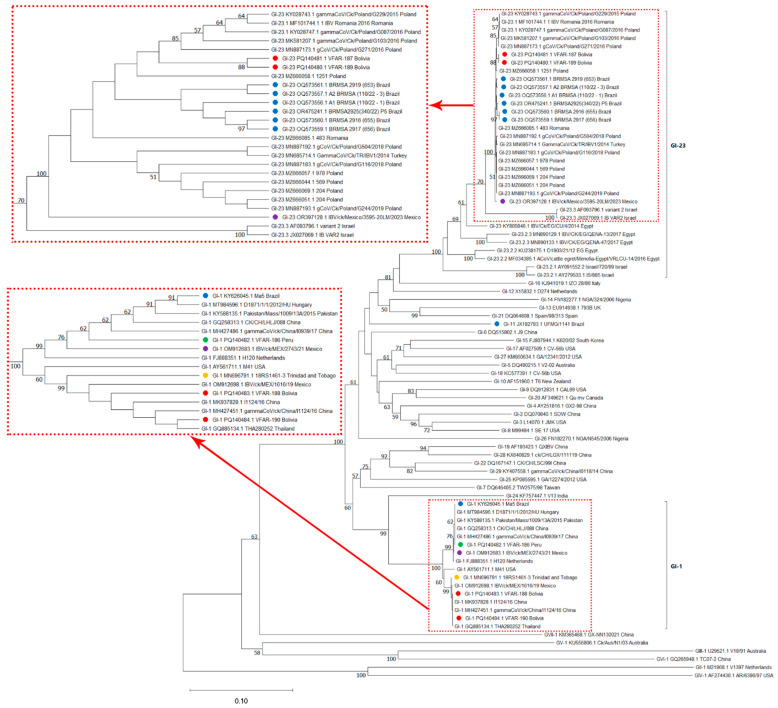
Phylogenetic tree based on full S1 gene sequences of Infectious Bronchitis Virus. The diagram was constructed using the Neighbor-Joining method with 1000 bootstrap replicates in MEGA version 11.0.13. The tree illustrates the evolutionary relationships among various IBV isolates, highlighting the GI-1 and GI-23 lineages. South American strains are indicated with different colored dots as follows: Bolivian strains (red), Brazilian strain (blue), Mexican strain (purple), Trinidad and Tobago strain (yellow), and Peruvian strain (green). Branch lengths correspond to evolutionary distances calculated using the Tajima–Nei method. The bootstrap support for each cluster is indicated on the respective branches.

The full S1 gene sequences generated from the four IBV samples identified from Bolivia were aligned and compared with 78 full S1 sequences representing various IBV lineages and genotypes, described by Valastro et al. (2016) [13], as well as sequences recently identified in Brazil [2]. The phylogenetic analysis revealed that all samples belonged to IBV genotype I (GI). Specifically, VFAR-188 and VFAR-190 were classified within the IBV GI-1 lineage, showing high sequence identity to the Massachusetts and H120 vaccine strains (>99%). In contrast, VFAR-187 and VFAR-189, collected from the same farm, belonged to the IBV GI-23 lineage, forming a distinct subclade that is genetically distant from Brazilian strains [9,32] (Figure 2). An initial estimate suggested that VFAR-187 and VFAR-189 were probably introduced into Bolivia. No evidence of recombination was detected. The recombination analysis indicated that strains FAR-187 and VFAR-189 did not undergo recombination events, clustering consistently with reference strains of the GI-23 lineage. In contrast, VFAR-188 and VFAR-190 grouped with vaccine-like strains.

### 3.3. Amino Acid Substitution Analysis

**Figure 3:**
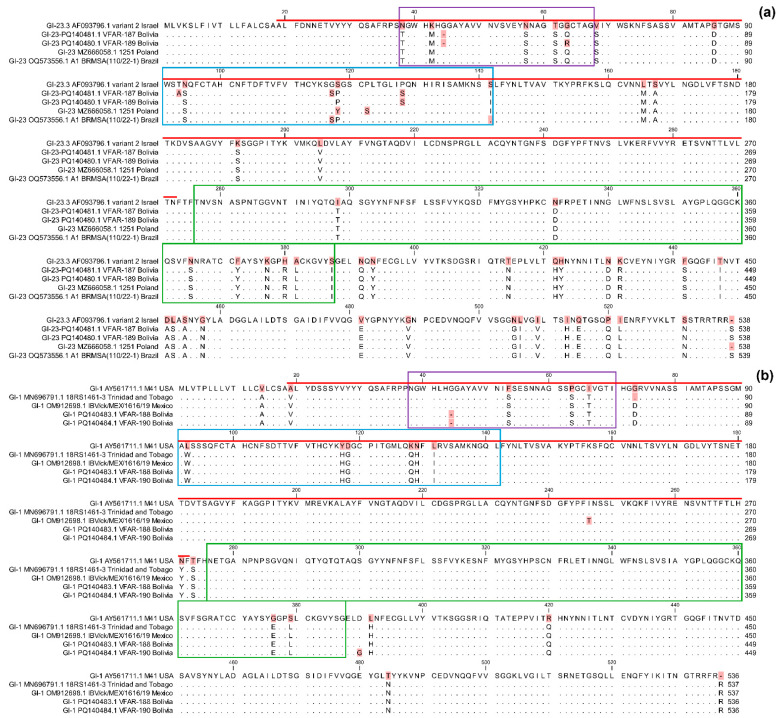
Comparative analysis of amino acid changes detected in the S1 gene of Infectious Bronchitis Virus strains belonging to the GI-1 and GI-23 lineages. HVR1 (purple), HVR2 (blue), and HVR3 (green) in the S1 gene amino acid sequence were compared among the Bolivian isolates and a representative strain and the closest relatives of each GI-1 and GI-23 lineage. (**a**) Comparison of the Bolivian VFAR-187 and VFAR-189 strains (lineage GI-23) with the Israeli strain (reference strain) and other related strains (Poland and Brazil). (**b**) Comparison of the Bolivian VFAR-188 and VFAR-190 strains (GI-1 lineage) with the M41 vaccine strain from the USA (reference strain) and other related strains from Trinidad and Tobago and Mexico. Amino acid changes are represented by different letters; those conserved are presented with dots, and deletions are presented with dashes. The receptor-binding domain (RBD) is indicated with a red line.

In the GI-23 lineage, several amino acid changes were identified in the Bolivian VFAR-187 and VFAR-189 strains within the S1 gene HVRs of the Infectious Bronchitis Virus, compared to the Israeli reference strain (AF093796.1) and the closest strains from Poland and Brazil. Specifically, both Bolivian strains exhibit a unique deletion at position G44 in HVR1 and a substitution at P128S in HVR2. Shared substitutions between the Bolivian, Polish, and Brazilian strains were observed, including N38T, K42M, N57S, and T61S in HVR1, N94S in HVR2, and I298T, N331D, N365S, F372Y, K377N, H380R, A381L, and S387I in HVR3. Notable differences were also identified between the Bolivian strains and the other strains. For instance, the mutation G63Q in HVR1 was found in both VFAR-187 and strains from Poland and Brazil, while the G117S mutation in HVR2 was shared between VFAR-187 and the Brazilian strain. Additionally, the S118P and S141I mutations in HVR2 were common to both Bolivian strains (VFAR-187 and VFAR-189) and either the Brazilian or Polish strains, respectively. Unique mutations were also observed, such as T93A in HVR2 (exclusive to the VFAR-187 strain) and G63R in HVR1 (exclusive to VFAR-189) (Figure 3a).

For the GI-1 lineage, the Bolivian VFAR-188 and VFAR-190 strains showed a unique deletion at G44 in HVR1 compared to the M41 vaccine strain from USA and the other closest strains. Additionally, shared substitutions between strains from Bolivia, Trinidad and Tobago, and Mexico were found, including F53S, P63S, and I66T in HVR1, L92W, Y117H, D118G, K128Q, N129H, and L131I in HVR2, and G376E and S379L in HVR3. No unique mutations were observed in either of the two Bolivian strains (Figure 3b).

## 4. Discussions

Infectious Bronchitis is a poultry disease caused by IBV that has been reported worldwide. Despite the availability of vaccines, the virus is constantly evolving, creating new variants. The emergence of new variants makes it challenging to protect chickens, as vaccines for one virus type may be ineffective against others [5]. Therefore, identifying the diversity of IBV viruses circulating in a country is crucial to improving protection measures. It is necessary to develop adequate and effective methods to study the genetic variability and evolution of viruses through their characterization.

In this study, we report for the first time the presence of IBV in a poultry farm in Cochabamba, Bolivia, confirming the circulation of genotype I (GI). Phylogenetic analyses of the full S1 gene sequence are crucial for differentiating and classifying IBV genotypes and lineages, serving as the gold standard for this purpose [13]. Based on full S1 phylogenetic analyses, we found that two Bolivian strains, VFAR-188 and VFAR-190, clustered within the GI-1 lineage, which predominantly comprises vaccine-like strains. Importantly, the GI-1 lineage represents the first identified IBV serotype and remains one of the most well-known and widely distributed genetic groups [13]. VFAR-188 and VFAR-190, identified in sick birds with respiratory symptoms, showed high similarity with the Massachusetts and H120 vaccine strains. This suggests that these two Bolivian strains may be derived from a vaccine-like strain. However, the birds were only administered one vaccine dose, which raises concerns, since they later developed respiratory symptoms. Live vaccines can sometimes become harmful in a process known as reversion to virulence [46], and therefore do not fully protect against different types of IBV or prevent disease outbreaks [16,47]. However, until now, the GI-1 lineage has been primarily derived from the live Massachusetts vaccine strain and is used in immunization programs throughout South America [9,48]. It is believed that many of the currently circulating Massachusetts viruses in South America originated as a result of these vaccines reverting to their original wild form [29]. Our findings support this hypothesis.

The Massachusetts strain of the Infectious Bronchitis Virus has been present in South America since the 1950s [49]. This strain, often used in vaccines, has become widespread across the region. Studies similar to ours carried out in Brazil, Colombia, Trinidad, Argentina, and Uruguay have all identified this vaccine-like strain as a common cause of IBV infections [2,20,21,29]. Coupled with our Bolivian results, these findings demonstrate a heterogeneous distribution of GI-1 in South America. We believe this is likely due to the potential reversion of live-attenuated vaccines [46], variations in disease control practices, and limitations in developing more effective vaccination strategies.

The full S1 gene sequences analysis demonstrated that the other two strains (VFAR-187 and VFAR-189) were clustered within the GI-23 lineage, forming a distinct subclade that is genetically distant from Brazilian strains. It is assumed that the VFAR-187 and VFAR-189 strains were probably introduced in Bolivia from elsewhere. Belonging to the GI-23 lineage, IBV is highly pathogenic and has been circulating since 1998 in the Middle East [2,50]. Over time, this lineage has spread across other continents, including Asia, Europe, and Africa [2,32]. In South America, the first detection of GI-23 was recorded in Brazil in 2022 [9,50] and has since spread throughout the country, causing upper respiratory tract and severe kidney lesions [2,9]. VFAR-187 strain exhibited unique characteristics, including macroscopic lesions in the trachea composed of mucus and congestion. Additionally, significant interstitial inflammatory renal lesions were observed in the VFAR-189 strain, suggesting that this strain has a strong tropism for the renal tract. These common features are specific to the highly pathogenic GI-23 lineage [2], and this finding is consistent with the results of a study by Lisowska et al., 2021 [31].

Three sublineages have been identified within the GI-23 lineage: 23.1, 23.2, and 23.3 [32]. Furthermore, the GI-23.2 sublineage is divided into three subclades: GI-23.2.1, GI-23.2.2, and GI-23.2.3 [32]. However, the phylogenetic analysis results indicate that the VFAR-187 and VFAR-189 strains do not seem to be closely related to the GI-23 subclades, suggesting that the Bolivian VFAR-187 and VFAR-189 strains could be clearly classified into other subclades or that they may possibly form another sublineage. Moreover, no evidence of recombination events giving rise to the Bolivian GI-23 strains was found. For this reason, we suggest that more samples coming from different regions of Bolivia should be analyzed.

S1 amino acid sequences from the Bolivian VFAR-187 and VFAR-189 strains presented specific and characteristic substitutions in the hypervariable regions (HVR1, HVR2, and HVR3). Amino acid differences in the S1 gene, particularly within the HVRs, are significant due to their potential impact on the antigenicity, tropism, and pathogenicity of Infectious Bronchitis Virus. HVRs are binding sites for neutralizing antibodies, and variations within these regions could affect the immune system’s ability to recognize and neutralize the virus [12]. Specifically, substitutions at positions 38, 43, 56, 63, 66, and 69 within HVR1 have been identified as critical for IBV attachment to chicken respiratory tract tissues [19,51]. In this study, we observed that the VFAR-187 and VFAR-189 strains have substitutions at positions N38T and G63Q/R within HVR1. These substitutions could potentially alter the ability of these variants to bind to respiratory tract tissue and cause disease. Additionally, substitutions in other positions within HVR2, particularly at positions T93A, N94S, G117S, S118P, P128S, and S141I, might also influence the antigenicity of VFAR-187 and VFAR-189 strains. These variations could result in a decreased efficacy of existing vaccines, which are generally based on reference strains such as the Massachusetts strain (GI-1). Furthermore, additional mutations identified in HVR3, such as I298T, N331D, N365S, F372Y, K377N, H380R, A381L, and S387I, could significantly impact the virus’s interaction with the immune system and its pathogenesis; however, to confirm this, more studies should be carried out.

On the other hand, the S1 amino acid sequences of the Bolivian VFAR-188 and VFAR-190 strains belonging to the GI-1 lineage showed a high degree of similarity to the M41 vaccine strain from the USA and two other close strains (Mexico and Trinidad and Tobago), mainly in HVR3. The substitution at position 63 in HVR1 exhibited by both Bolivian strains could also be related to the ability of IBV to adhere to the chicken respiratory tract and cause disease [19]. Additionally, the unique deletion at position G44 in HVR1 observed in the VFAR-188 and VFAR-190 strains suggests the existence of possible adaptations that could influence the virus’s ability to evade the immunity conferred by current vaccines.

The presence of the IBV GI-23 lineage in Bolivia is a recent discovery, and it is not yet fully understood how it arrived in the country. The phylodynamic characteristics of the IBV GI-23 lineage are still uncertain; however, we can assume that its presence in Bolivia could be related to the poultry trade and the movement of people between countries, despite the biosecurity measures implemented. For this reason, future studies should focus on isolating and characterizing IBV from both vaccinated and unvaccinated flocks in Bolivian farms to identify potential vaccine-like or recombinant strains causing disease. Additionally, experimental infection studies are necessary to evaluate the efficacy of current vaccines against the GI-23 variant circulating in Bolivian commercial poultry farms. It is essential to investigate whether the GI-23 variant was recently introduced in Bolivia or if it has been circulating undetected.

## 5. Conclusions

This study provides the first evidence of the presence of the IBV lineages GI-1 and GI-23 in a high-density avian region in Bolivia, marking a significant finding in the epidemiology of IBV in South America. The clustering of two Bolivian strains within the GI-1 lineage, with strong clade support for vaccine-like strains, indicates a possible reversion to virulence, highlighting the importance of monitoring and managing vaccine strains. The identification of GI-23 in Bolivia, a lineage known for its pathogenicity and global presence, raises concerns about its potential impact on poultry health and the effectiveness of existing vaccines in the region.

The amino acid substitution analysis identified specific mutations in the S1 subunit, particularly in the hypervariable regions 1, 2, and 3. These mutations could potentially alter the virus’s antigenicity, leading to reduced vaccine efficacy.

To further understand the implications of these findings, it is crucial to conduct additional studies focusing on the isolation and characterization of IBV strains from both vaccinated and unvaccinated flocks in Bolivia. Experimental infection studies are also needed to assess the effectiveness of current vaccines against the circulating field strains. Finally, investigating the origin and spread of the GI-23 variant in Bolivia will be vital in developing strategies to control and prevent IBV outbreaks in the region.

## Figures and Tables

**Table 1:** Bolivian samples used in this study.

Sample	Sequencing ID	Sample Type	Clinical Signs	Age	Year	Country of Origin	RT-qPCR Ct Value
524	na	Trachea	Respiratory	21 days	2024	Bolivia	+/25.83
525	VFAR-187	Trachea	Respiratory and trachea lesions	21 days	2024	Bolivia	+/23.73
526	na	Paranasal sinuses	Respiratory	21 days	2024	Bolivia	+/27.00
527	na	Kidney	Nephritis	21 days	2024	Bolivia	−/no Ct
529	VFAR-188	Trachea	Respiratory	28 days	2024	Bolivia	+/19.26
530	VFAR-189	kidney	Respiratory and nephritis	25 days	2024	Bolivia	+/23.08
531	VFAR-190	Paranasal sinuses	Respiratory	28 days	2024	Bolivia	+/18.93
532	na	Kidney	Nephritis	25 days	2024	Bolivia	−/no Ct
534	na	Trachea	Respiratory	33 days	2024	Bolivia	+/26.10
535	na	Cecal tonsil	Respiratory and diarrhea	33 days	2024	Bolivia	+/30.15
536	na	Paranasal sinuses	Respiratory	33 days	2024	Bolivia	+/29.86
537	na	Kidney	Nephritis	33 days	2024	Bolivia	+/31.92

na: not applicable, +: positive sample, −: negative sample.

## Data Availability

The data presented in this study are openly available in GenBank with the numbers PQ140481.1, PQ140483.1, PQ140480.1, and PQ140484.1.

## Supplementary Materials

The following supporting information can be downloaded at https://www.mdpi.com/article/10.3390/v16091463/s1, Table S1: GenBank reference sequences used in the S1 gene sequence alignment and phylogenetic analysis.
