# Supplementary material for: Detection and Molecular Characterization of GI-1 and GI-23 Avian Infectious Bronchitis Virus in Broilers Indicate the Emergence of New Genotypes in Bolivia
Source: Viruses. 2024 Sep 14;16(9):1463. doi: 10.3390/v16091463 (PMC11437422; doi:10.3390/v16091463)
Supplement: Supplementary file 1 [file viruses-16-01463-s001.zip › viruses-3192327-supplementary.pdf]

**Table S1.** GenBank reference sequences used in the S1 gene sequence alignment and phylogenetic analysis.

| Strain Name | Collection Year | Country of Origin | Genotype and Lineage | GenBank Accession Number |
|---|---|---|---|---|
| H120 | 1960 | The Netherlands | GI-1 | FJ888351 |
| M41 | 1956 | USA | GI-1 | AY561711.1 |
| Ma5 | 2016 | Brazil | GI-1 | KY626045.1 |
| D1871/1/1/2012/HU | 2012 | Hungary | GI-1 | MT984596.1 |
| Pakistan/Mass/1009/13A/2015 | 2015 | Pakistan | GI-1 | KY588135.1 |
| CK/CH/LHLJ/08II | 2008 | China | GI-1 | GQ258313.1 |
| gammaCoV/ck/China/I0939/17 | 2017 | China | GI-1 | MH427486.1 |
| IBV/ck/MEX/2743/21 | 2021 | Mexico | GI-1 | OM912683.1 |
| 18RS1461-3 | 2014 | Trinidad and Tobago | GI-1 | MN696791.1 |
| IBV/ck/MEX/1616/19 | 2019 | Mexico | GI-1 | OM912698.1 |
| I1124/16 | 2016 | China | GI-1 | MK937828.1 |
| gammaCoV/ck/China/I1124/16 | 2016 | China | GI-1 | MH427451.1 |
| THA280252 | 2009 | Thailand | GI-1 | GQ885134.1 |
| SDW | 2005 | China | GI-2 | DQ070840.1 |
| JMK | 1993 | USA | GI-3 | L14070.1 |
| GX2-98 | 2003 | China | GI-4 | AY251816.1 |
| V2-02 | 2006 | Australia | GI-5 | DQ490215.1 |
| J9 | 2006 | China | GI-6 | DQ515802.1 |
| TW2575/98 | 2008 | Taiwan | GI-7 | DQ646405.2 |
| SE 17 | 1993 | USA | GI-8 | M99484 |
| CAL99 | 1999 | California/USA | GI-9 | DQ912831.1 |
| T6 S1 | 1999 | New Zealand | GI-10 | AF151960.1 |
| UFMG/1141 | 2009 | Brazil | GI-11 | JX182783.1 |
| D274 | 1989 | The Netherlands | GI-12 | X15832.1 |
| Moroccan-G/83 | 1983 | Morocco | GI-13 | EU914938.1 |
| NGA/324/2006 | 2006 | Nigeria | GI-14 | FN182277.1 |
| K620/02 | 2002 | South Korea | GI-15 | FJ807944.1 |
| IZO 28/86 | 1986 | Italy | GI-16 | KJ941019.1 |
| CV-56b | 1997 | USA | GI-17 | AF027509.1 |
| 53XJ-99II | 1999 | China | GI-18 | KC577391.1 |
| QXIBV | 1999 | China | GI-19 | AF193423.1 |
| Qu_mv | 1996 | Canada | GI-20 | AF349621.1 |
| Spain/98/313 | 2008 | Spain | GI-21 | DQ064808.1 |
| CK/CH/LSC/99I | 2006 | China | GI-22 | DQ167147.1 |
| IBV/Ck/EG/CU/4/2014 | 2014 | Egypt | GI-23 | KY805846.1 |
| 1251 | 2020 | Poland | GI-23 | MZ666058.1 |
| A1 BRMSA (110/22 - 1) | 2022 | Brazil | GI-23 | OQ573556.1 |
| 978 | 2019 | Poland | GI-23 | MZ666057.1 |
| GammaCoV/Ck/TR/IBV1/2014 | 2014 | Turkey | GI-23 | MN685714.1 |
| 569 | 2018 | Poland | GI-23 | MZ666044.1 |
| gCoV/Ck/Poland/G244/2019 | 2019 | Poland | GI-23 | MN887193.1 |
| gammaCoV/Ck/Poland/G103/2016 | 2016 | Poland | GI-23 | MK581207.1 |
| 483 | 2019 | Romania | GI-23 | MZ666085.1 |
| 204 | 2018 | Poland | GI-23 | MZ666069.1 |
| 834 | 2019 | Poland | GI-23 | MZ666051.1 |
| BRMSA2925(340/22) P5 | 2022 | Brazil | GI-23 | OR475241.1 |
| gCoV/Ck/Poland/G504/2018 | 2018 | Poland | GI-23 | MN887192.1 |
| BRMSA 2919 (653) | 2021 | Brazil | GI-23 | OQ573561.1 |
| BRMSA 2916 (655) | 2021 | Brazil | GI-23 | OQ573560.1 |
| BRMSA 2917 (656) | 2021 | Brazil | GI-23 | OQ573559.1 |
| gammaCoV/Ck/Poland/G229/2015 | 2015 | Poland | GI-23 | KY028743.1 |
| 3595-20LM/23 | 2023 | Mexico | GI-23 | OR397128.1 |
| gCoV/Ck/Poland/G271/2016 | 2016 | Poland | GI-23 | MN887173.1 |
| gCoV/Ck/Poland/G116/2018 | 2018 | Poland | GI-23 | MN887183.1 |

| | | | | |
|---|---|---|---|---|
| A2 BRMSA (110/22 - 3) | 2022 | Brazil | GI-23 | OQ573557.1 |
| variant 2 | 1998 | Israel | GI-23.3 | AF093796.1 |
| IB VAR2 | 2012 | Israel | GI-23.3 | JX027069.1 |
| gammaCoV/Ck/Poland/G087/2016 | 2016 | Poland | GI-23.1 | KY028747.1 |
| 1_IBV_Romania_2016 | 2016 | Romania | GI-23.1 | MF101744.1 |
| Israel/720/99 | 2002 | Israel | GI-23.2.1 | AY091552.2 |
| IS/885 | 2003 | Israel | GI-23.2.1 | AY279533.1 |
| D1903/21/12_EG | 2012 | Egypt | GI-23.2.2 | KU238175.1 |
| ACoV/cattle egret/Menofia-Egypt/VRLCU-14/2016 | 2016 | Egypt | GI-23.2.2 | MF034385.1 |
| IBV/CK/EG/QENA-13/2017 | 2017 | Egypt | GI-23.2.3 | MN890129.1 |
| IBV/CK/EG/QENA-47/2017 | 2017 | Egypt | GI-23.2.3 | MN890133.1 |
| V13 | 1998 | India | GI-24 | KF757447.1 |
| GA/12274/2012 | 2012 | USA | GI-25 | KP085595.1 |
| NGA/N545/2006 | 2006 | Nigeria | GI-26 | FN182270.1 |
| GA/12341/2012 | 2012 | USA | GI-27 | KM660634.1 |
| ck/CH/LGX/111119 | 2011 | China | GI-28 | KX640829.1 |
| gammaCoV/ck/China/I0118/14 | 2011 | China | GI-29 | KY407558.1 |
| V1397 | 1989 | The Netherlands | GII-1 | M21968.1 |
| V18/91 | 1996 | Australia | GIII-1 | U29521.1 |
| AR/6386/97 | 2000 | USA | GIV-1 | AF274436.1 |
| Ck/Aus/N1/03 | 2003 | Australia | GV-1 | KU556806.1 |
| TC07-2 | 2007 | China | GVI-1 | GQ265948.1 |
| GX-NN130021 | 2013 | China | GVII-1 | KM365468.1 |
| VFAR-186 | 2024 | Peru | GI-1 | PQ140482.1 |
| VFAR-187* | 2024 | Bolivia | GI-23 | PQ140481.1 |
| VFAR-188* | 2024 | Bolivia | GI-1 | PQ140483.1 |
| VFAR-189* | 2024 | Bolivia | GI-23 | PQ140480.1 |
| VFAR-190* | 2024 | Bolivia | GI-1 | PQ140484.1 |

* Samples sequenced in this study.
